# Bio- chemical and physical characterizations of mesenchymal stromal cells along the time course of directed differentiation

**DOI:** 10.1038/srep31547

**Published:** 2016-08-16

**Authors:** Yin-Quan Chen, Yi-Shiuan Liu, Yu-An Liu, Yi-Chang Wu, Juan C. del Álamo, Arthur Chiou, Oscar K. Lee

**Affiliations:** 1 Institute of Biophotonics, National Yang-Ming University, Taipei 11221, Taiwan; 2 Biophotonics & Molecular Imaging Research Center, National Yang-Ming University, Taipei 11221, Taiwan; 3 Stem Cell Research Center, National Yang-Ming University, Taipei 11221, Taiwan; 4 Department of Mechanical and Aerospace Engineering, University of California San Diego, La Jolla, CA 92093, USA; 5 Institute for Engineering in Medicine, University of California San Diego, La Jolla, CA 92093, USA; 6 Taipei City Hospital, Taipei 10341, Taiwan; 7 Institute of Clinical Medicine, National Yang-Ming University, Taipei 11221, Taiwan; 8 Department of Medical Research, Taipei Veterans General Hospital, Taipei 11217, Taiwan

## Abstract

Cellular biophysical properties are novel biomarkers of cell phenotypes which may reflect the status of differentiating stem cells. Accurate characterizations of cellular biophysical properties, in conjunction with the corresponding biochemical properties could help to distinguish stem cells from primary cells, cancer cells, and differentiated cells. However, the correlated evolution of these properties in the course of directed stem cells differentiation has not been well characterized. In this study, we applied video particle tracking microrheology (VPTM) to measure intracellular viscoelasticity of differentiating human mesenchymal stromal/stem cells (hMSCs). Our results showed that osteogenesis not only increased both elastic and viscous moduli, but also converted the intracellular viscoelasticity of differentiating hMSCs from viscous-like to elastic-like. In contrast, adipogenesis decreased both elastic and viscous moduli while hMSCs remained viscous-like during the differentiation. In conjunction with bio- chemical and physical parameters, such as gene expression profiles, cell morphology, and cytoskeleton arrangement, we demonstrated that VPTM is a unique approach to quantify, with high data throughput, the maturation level of differentiating hMSCs and to anticipate their fate decisions. This approach is well suited for time-lapsed study of the mechanobiology of differentiating stem cells especially in three dimensional physico-chemical biomimetic environments including porous scaffolds.

Mesenchymal stromal/stem cells (MSCs) are adult stem cells of stromal origin capable of self-renewal and directed differentiation into diverse specialized cell types^1^. With immunomodulatory properties and low immunogenicity, multipotent MSCs provide a great potential in tissue engineering for regenerative medicine^2^. However, efficient and precise directed differentiation of MSCs into specific functional cell types remains challenging. In addition to growth factors and cytokines that act as chemical cues for regulating stem cell differentiation, accumulated studies have demonstrated that physical properties of the microenvironments can act as mechanical cues to modulate the fate commitments as well^3^,^4^. A better understanding of the interplay between the biochemical and the biophysical cues during differentiation process could improve the efficiency for directed differentiation. Cells generate contractile forces and rearrange their cytoskeletal network in response to environmental mechanical stimuli. Thus, changes in biophysical parameters, such as cell shape^5^,^6^, cytoskeletal organization^7^,^8^,^9^, and intracellular viscoelastic properties can be used as early markers of the effect of mechanical stimulation on MSC fate commitment^10^. However, the changes in biophysical properties along the time-course of MSC differentiation are yet to be characterized.

Several platforms have been developed to probe the viscoelastic properties of MSCs in the early or late stages of differentiation at single cell level, including atomic force microscopy (AFM)^11^,^12^,^13^,^14^, micropipette aspiration^15^,^16^, optical tweezers^13^,^17^, and video particle tracking microrheology (VPTM)^18^. AFM systems equipped with a sharp tip^19^ have been shown to probe local cell stiffness caused by the interaction between cortex actin and cell membrane, whereas those equipped with colloidal force probe^20^,^21^ have been demonstrated to analyze global cell stiffness. Likewise, micropipette aspiration provides global measures of whole-cell stiffness, while optical tweezers can provide either local or global measurement depending on the optical configurations^13^,^17^. VPTM measures the local viscoelastic response of the cytoplasm^22^ despite the fact that the motion of VPTM probing particles may be restricted by nearby organelles and complex membrane structures (e.g. the endoplasmatic reticulum)^23^,^24^,^25^. Furthermore, it can be extended to determine the viscoelastic response along different directions in cells with preferential cytoskeletal fiber alignment^26^.

VPTM has two key merits compared to other techniques for measuring mechanical properties of living cells such as AFM, micropipette aspiration or optical tweezers. Firstly, it can be used in living cells embedded in 3-dimensional extracellular matrix (3-D ECM) as long as the probing particles are injected in the cells prior to 3D culture. For example, an oil immersion objective (Nikon S Fluor, 100X, NA = 1.3) with long working distance (WD = 0.2 mm) can be used to image and track the motion of the particles embedded in cells seeded in a thick (~70 to 100 μm) 3-D scaffold and/or extracellular matrix above a coverslip (with a thickness of 0.10 to 0.13 mm). Secondly, the data throughput of VPTM is higher than that of AFM, micropipette aspiration or optical tweezers, as explained in the materials and methods section.

In this study, we systematically measured biophysical parameters, including cell morphology, size of focal adhesion complex, actin arrangement, and intracellular viscoelasticity, during osteogenic and adipogenic differentiations of human MSCs (hMSCs) up to 28 days. We complemented these parameters with biochemical parameters along the time course of differentiation, including expression of differentiation genes, cytoskeleton related genes, and focal adhesion related genes. Our results reveal that a hyper-dimensional representation of these parameters along the time-course of differentiation process may provide an overall view of how these parameters evolve quantitatively in parallel. We further deduced from these data that during osteogenic differentiation of hMSCs a strong positive correlation (with Pearson Correlation Coefficient PCC = 0.86) exists between the magnitude of complex shear modulus (|G*|) and the gene expression of Collagen type1 alpha1 (COL1A1). In contrast, there is a strong negative correlation (PCC = −0.94) between |G*| and the gene expression of CCAAT-enhancer-binding proteins (C/EBP) during adipogenic differentiation.

## Results

### Gene expression profiles of MSCs validated the differentiation processes during osteogenesis and adipogenesis

**Figure 1:**
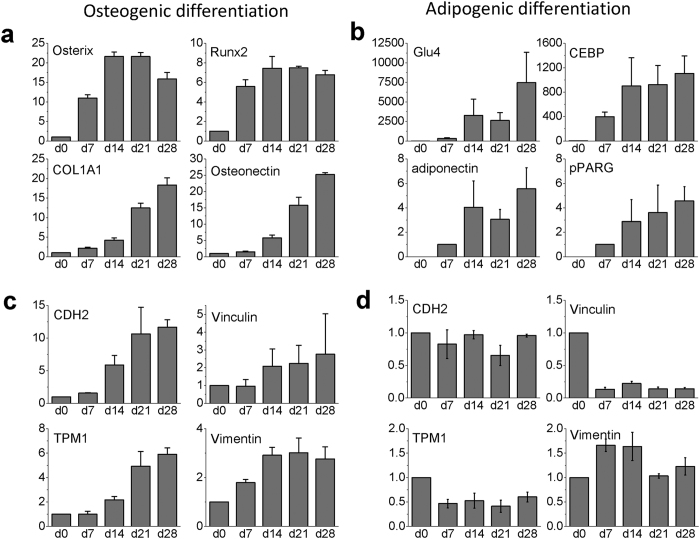
Gene expression profiling of hMSCs in osteogenic and adipogenic differentiations from day 0 (d0) to day 28 (d28) measured at 7-day interval. (**a**) Relative expressions of osteogenic marker genes Osterix (SP7), Runx2, collagen type 1 alpha1, osteonectin during osteogenesis, and (**b**) adipogenic marker genes Glu4, adiponectin, PPARG, C/EBP during adipogenesis of hMSCs were upregulated. (**c**) Relative expressions of adhesion related genes CDH2, Vinculin and cytoskeleton related genes TPM1, Vimentin were upregulated in osteogenic differentiation. (**d**) In adipogenic differentiation, vinculin and TPM1 were downregulated; expressions of CDH2 and vimentin remained at the basal level. Gene expressions were analyzed by RT-PCR. Data were normalized by the respective gene expressions of undifferentiated MSCs (day 0) and presented as mean ± SD (N = 3), excepted for the cases of adiponectin and PPARG in (**b**) where the data were normalized by the corresponding values on day 7 because the values on day 0 was below our detection limit.

The degree of maturation of directed differentiation was first assessed by quantitative real time PCR of hMSCs cultured on glass bottom with collagen coating. Osterix (SP7) and Runt-related transcription factor 2 (RUNX2) are two essential transcription factors for osteoblast differentiation. Collagen type1 alpha1 (COL1A1) and osteonectin (SPARC) are two marker proteins associated with bone formation. These four genes were upregulated after osteogenic induction, with genes of the transcription factors reaching maximum expression on day 14 or day 21 and gene expressions for the osteogenic marker proteins gradually increasing till day 28 after the induction (Fig. 1a). Similarly, expressions of adipogenic related genes adiponectin, peroxisome proliferator-activated receptor gamma (PPARG), solute carrier family 2 member 4 (GLU4), and CCAAT-enhancer-binding proteins (C/EBP) were upregulated after adipogenic induction (Fig. 1b). The results of gene expressions demonstrated that differentiations by chemical inductions were incomplete on day 7 but gradually reached the maturations on day 28, which is consistent with our previous studies of hMSCs cultured on Petri dishes^1^.

We also investigated the gene expressions of adhesion related genes N-cadherin (CDH2) and Vinculin, as well as cytoskeletal related genes Tropomyosin alpha-1 chain (TPM1) and Vimentin during osteogenesis and adipogenesis (Fig. 1c,d). All of these four genes gradually increased in osteogenic differentiation, but either decreased or remained at the basal levels in adipogenic differentiation.

### Differentiation proceeded as the cell morphology changed from highly-elongated to more-rounded along with a large increase in cell area

**Figure 2:**
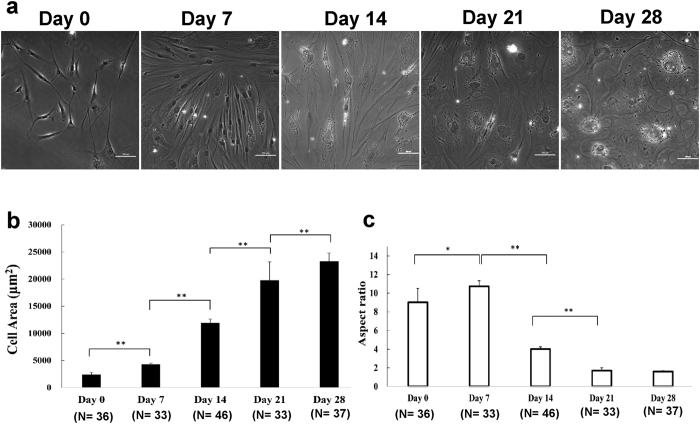
Changes of cellular morphological during the time course of osteogenic differentiation. **(a)** The phase contrast images of hMSCs at different stages of osteogenic differentiation from day 0 and day 28 (recorded at 7-day interval). Scale bars = 100 μm. **(b)** Cell spreading area and **(c)** aspect ratio (defined as the ratio of the length of major and minor axes) at different stages of osteogenic differentiation of hMSCs. The mean values and standard error of the mean (SEM) are indicated by the height of the thick bars and the thin lines. *p < 0.05; **p < 0.01 (by Student’s t-test).

Phase contrast micrographs of hMSCs at different stages of osteogenic differentiation showed that the cells were highly elongated in early stages from day 0 to day 14, and became more spread and rounded in later stages after day 21 (Fig. 2a). The spreading area of MSCs increased monotonically by a factor of 10 from 2.3 × 10^3^ μm^2^ at day 0 to 2.3 × 10^4^ μm^2^ at day 28 during osteogenesis (Fig. 2b). We further quantified the morphological changes in terms of the cell spreading area and the aspect ratio, defined as the ratio of the length of major to minor axes (Fig. 2c). The changes in cell morphology (i.e., cell area and aspect ratio) during adipogenic differentiation followed similar trends (Supplementary Fig. S1). These results show that, in general, the change in cellular area bears a positive correlation, while the aspect ratio bears a negative correlation with the expression of differentiation marker genes for osteogenic differentiation (Figs 1a,c and 2a,b) as well as adipogenic differentiation (Fig. 1b,d, and Supplementary Fig. S1). Hence, the changes in these morphological parameters could not serve to distinguish osteogenic differentiation against adipogenic differentiation.

### Intracellular viscoelastic moduli of hMSCs increased steadily to approach that of the human fetal osteoblast cells in osteogenic differentiation

During the course of osteogenic differentiation of hMSCs from day 0 to day 28, we measured, via video particle tracking microrheology (VPTM), the intracellular viscoelastic properties in terms of the elastic shear modulus G′(f), and the viscous shear modulus G″(f). These shear moduli were measured at 7-day intervals as functions of frequency f in the frequency range of 1 Hz to 100 Hz. For examples, our experimental results for MSD (mean square displacement) of the particles as a function of time-lag for hMSCs at day 0 is given in Supplementary Fig. S2, and the corresponding complex shear moduli as a function of frequency is given in Supplementary Fig. S3, which shows that both the elastic modulus *G*′(*f*) and the viscous modulus *G″*(*f*) followed a weak power-law with an exponent of 0.43 to 0.66 and 0.54 to 0.74, respectively.

**Figure 3:**
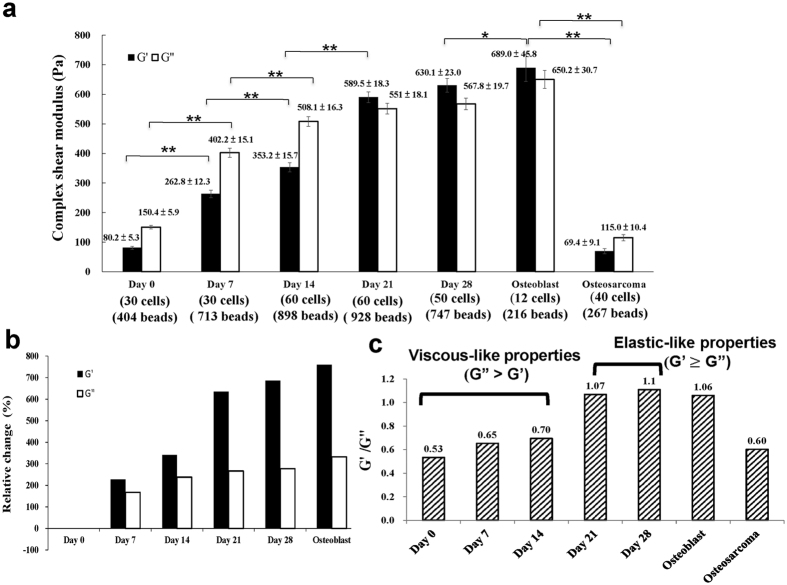
Increased intracellular viscoelasticity of hMSCs during osteogenic differentiation. **(a)** The intracellular elastic modulus (G′) and viscous modulus (G″), both at 10 Hz, of hMSCs during osteogenic differentiation. For comparison, the corresponding values (measured by identical method) for human fetal osteoblast cells and osteosarcoma cells (MG63) are shown in the 2 pairs of bars in the far right. Error bars represent SEM (standard error of the mean). **(b)** The fractional change of elastic modulus (G′) and viscous modulus (G″) of hMSCs (at 10 Hz) during osteogenic differentiation. The fractional change (%) = [(G_N_ – G_0_)/G_0_] × 100, where G_0_ represents either the elastic or the viscous modulus of hMSCs at day 0, and G_N_ represents the corresponding value at different stages of differentiation. **(c)** The viscoelastic properties of hMSCs during osteogenic differentiation quantified in terms of the ratio of elastic modulus (G′) and viscous modulus (G″) (G′/G″ at 10 Hz). *p < 0.05; **p < 0.01.

Both the elastic modulus G′ and the viscous modulus G″ increased steadily from day 0 to day 28; the results at f = 10 Hz are shown in Fig. 3a. For comparison, the corresponding values for human fetal osteoblast cells and human bone osteosarcoma cells (MG63) are also shown in Fig. 3a,c, which reveal that the elastic and viscous moduli of osteoblasts were approximately 8-fold and 4-fold higher than the corresponding values of undifferentiated hMSCs, and similar to those of differentiated hMSCs on day 28. In sharp contrast, the elastic and viscous moduli of osteosarcoma cells were comparable to those of undifferentiated hMSCs on day 0.

The corresponding results at 1 Hz and 50 Hz (Supplementary Fig. S3) validate that such a trend is not specific to viscoelastic moduli at 10 Hz. Similar results observed when hMSCs were osteogenic induced at a high seeding density (Supplementary Fig. S4) show that such a trend is fairly insensitive to the seeding density. Additionally, G′ and G″ were not affected by cell density, and G′/G″ of undifferentiated MSCs with 40% to 100% confluency is around 0.5 (Fig. 3c and Supplementary Fig. S4).

Throughout this article, the frequency f = 10 Hz was selected as a convenient reference frequency to facilitate the comparison of G′ and G″, as inference of shear moduli from VPTM at lower frequencies may be complicated by active cellular processes^27^; besides, the S/N ratio in the high frequency ends of G′(*f*) and *G″*(*f*) were limited by the frame rate of our CMOS camera and possibly also by other factors.

The increase in shear moduli during the course of osteogenic differentiation was accompanied by a transition from a fluid-like to an elastic-like behavior in the cells. This transition occurred because the increase in the elastic modulus was much higher than the corresponding increase in viscous modulus (Fig. 3b). Consequently, hMSCs showed a more viscous-like (G′ < G″) mechanical behavior in the early stage of differentiation from day 0 to day 14, but a more elastic-like (G′ > G″) behavior in later stages from day 21 to day 28 (Fig. 3c). This overall tendency of increasing stiffness, along with the shift towards more elastic-like mechanical response (Fig. 3a–c), is consistent with the increasing expressions of cytoskeleton and adhesion related genes (Fig. 1c). Thus, the alteration of viscoelastic properties during osteogenic differentiating of hMSCs could serve to substantiate the degree of maturation assessed by the expression of osteogenic marker genes (Fig. 1a,c).

### Osteogenic differentiation of hMSCs induced actin rearrangement and increased focal adhesion size and number

**Figure 4:**
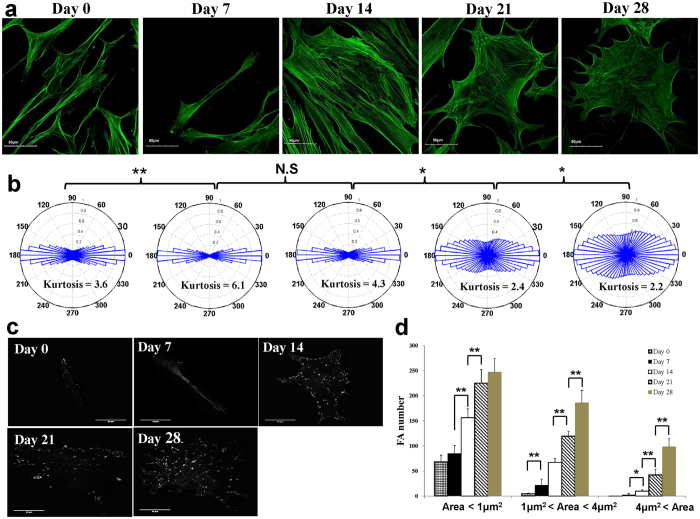
Actin rearrangement as well as focal adhesion size and number during osteogenic differentiation. **(a)** Immunofluorescence micrographs of actin filaments of hMSCs. The thin and long actin filaments were fairly well-aligned in the early stages, and became thicker and reoriented to assume a wide angular distribution during the course of osteogenic differentiation. Scale bars = 50 μm. **(b)** The angular distribution of actin filaments orientation at different stages of osteogenic differentiation of hMSCs. *p < 0.05; **p < 0.01. Data deduced from 15 cells. **(c)** Immunofluorescence micrographs of focal adhesions (FAs) in hMSCs. MSCs were stained with vinculin for the determination of FAs. Scale bars = 50 μm. **(d)** The number and size distribution of focal adhesions (FAs) in hMSCs at different stages of osteogenic differentiation. The quantitation of FAs was analyzed by MetaMorph software. All data are expressed as mean ± SD from 15 cells.

Via FITC-phalloidin staining, we recorded and analyzed actin images of hMSCs at different stages of osteogenic differentiation. As shown in Fig. 4a, the long and thin actin filaments of hMSCs were fairly well-aligned along the cells major axis at early stages and became thicker and reoriented to occupy a more uniform angular distribution as the cells assumed a more rounded shape and increased spreading area at later stages. These observations were quantitatively confirmed by the Kurtosis coefficient of the angular distributions in Fig. 4b, which decreased from day 7 to day 28 as the actin filaments adopted an increasingly wider angular distribution. As expected, the trend of the change in Kurtosis was consistent with that of the aspect ratio since the arrangement of actin cytoskeletal plays an important role in maintaining the cellular morphology.

The dynamic of focal adhesion (FA) formation is crucial in osteogenic differentiation^28^. In addition to being activated by chemical induction, osteogenic transcription factor Runx2 can be activated by FA-mediated extracellular signal-related kinase (ERK)^29^,^30^,^31^. To visualize the focal adhesions of hMSCs at different stages of osteogenic differentiation, hMSCs were stained with vinculin, a membrane-cytoskeletal protein forming focal adhesion complex (Fig. 4c). We found that in the course of osteogenic differentiation, not only the number of FA, but also the area of individual FA increased (Fig. 4d).

### Intracellular viscoelastic moduli of MSCs decreased steadily and no actin realignment was observed during adipogenesis

**Figure 5:**
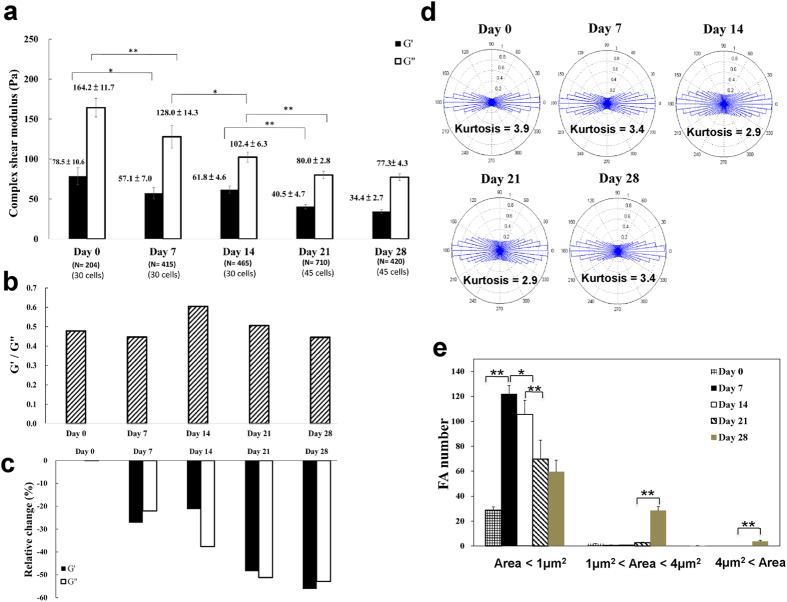
The biophysical properties of hMSCs at different stages of adipogenic differentiation. **(a)** The intracellular elastic modulus (G′) and viscous modulus (G″) (at 10 Hz) of hMSCs. Data are expressed as mean ± SEM (standard error of the mean). **(b)** The relative change of elastic modulus (G′) and viscous modulus (G″) of hMSCs (at 10 Hz). The relative change (%) = [(G_N_ – G_0_)/G_0_] × 100, where G_0_ represents either the elastic or the viscous modulus of hMSCs at day 0, and G_N_ represents the corresponding value at different stages of differentiation. **(c)** The viscoelastic properties of hMSCs quantified in terms of the ratio of elastic modulus (G′) and viscous modulus (G″) (G′/G″ at 10 Hz). **(d)** The angular distribution of actin filaments orientation. Data obtained from N = 15 cells. **(e)** The number and size distribution of focal adhesions (FAs) in hMSCs. The quantitation of FAs was analyzed by MetaMorph software. Data are expressed as mean ± SD. *p < 0.05; **p < 0.01. Data obtained from N = 15 cells.

We further characterized the biophysical properties, including the elastic modulus G′ and the viscous modulus G″, actin arrangement, as well as number and size distribution of focal adhesions of hMSCs during adipogenic differentiation. The results are shown in Fig. 5. In contrast to osteogenic differentiation, adipogenesis led to a decrease in both elastic and viscous shear moduli, G′ and G″, less of an effect on actin realignment and the cytoplasm remained fluid-like (G′ < G″). The smaller FAs (area < 1μm^2^) increased slightly from day 0 to day 7, followed by a steady decrease from day 7 to day 28 (Fig. 5e). As for total FA area, both osteogenic and adipogenic differentiations are accompanied by a steady increase in total FA area (Supplementary Fig. S5). However, in comparison with adipogenic differentiation, total FA area increased much more dramatically in the course of osteogenic differentiation.

Overall, we carried out each experiment (under identical conditions) with 3 independent biological replicates. The results for intracellular viscoelasticity of human MSCs during osteogenic and adipogenic differentiations are given in Supplementary Figs S6(a,b) and S7(a,b), respectively; each bar represents mean ± S.E.M (standard error of the mean) obtained from each replicate experiment with 30 cells per replicate. Likewise, the corresponding results (of 3 independent biological replicates) for cell area and morphological aspect ratio during the course of osteogenic and adipogenic differentiations are given in Supplementary Figs S6(c,d) and S7(c,d), respectively. In all cases, although there is a slight variation in the mean value obtained from each replicate experiment, the general trend in each case is quite consistent.

### Differentiation fate commitments were delineated cooperatively by biochemical and biophysical factors

**Figure 6:**
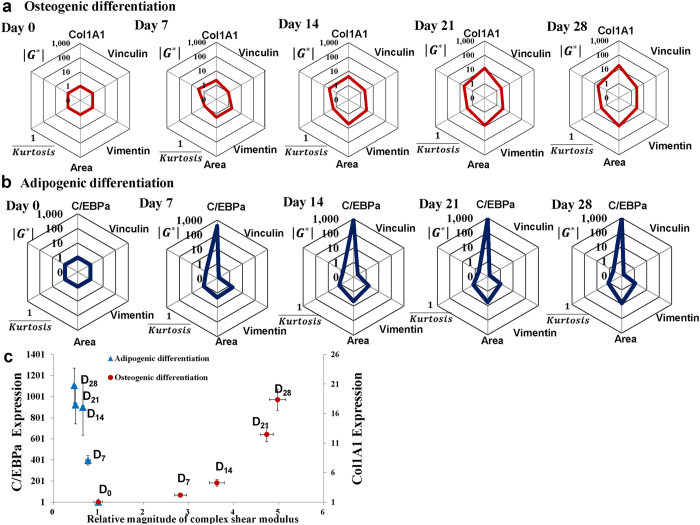
The biophysical and biochemical characterizations of hMSCs at different stages of osteogenic and adipogenic differentiation. The radar chart to present quantitatively the change in biophysical and biochemical properties in hMSCs at different stages of **(a)** osteogenic differentiation and **(b)** adipogenic differentiation from day 0 to day 28. The 6 parameters, namely, cell spreading area, elastic modulus (G′), viscous modulus (G″), aspect ratio, and kurtosis are presented along the 6 corner of the hexagon, with the first regular hexagon representing their initial values (defined as unity for undifferentiated hMSCs at day 0), and the subsequent irregular hexagons representing the value of each parameter (normalized by the corresponding values at day 0). **(c)** Scatter plots of relative differentiation marker genes and relative magnitude of complex shear modulus (|G*|) of hMSCs at different stages of osteogenic and adipogenic differentiation (normalized to the value of the corresponding parameter at day 0).The relative C/EBPα expression (adipogenic marker gene) and Col1A1 expression (osteogenic marker gene) refer to the left and the right vertical axes, respectively.

To visualize how the biophysical and biochemical parameters evolved during the different stages of osteogenic and adipogenic differentiation, we presented them together on a hexagonal parametric space that includes cell spreading area, magnitude of complex shear modulus (|G*|), 1/kurtosis of MSCs and gene expressions (Fig. 6a,b). A quick glance at Fig. 6a,b clearly reveals that the time development of osteogenic and adipogenic differentiations differ significantly in these hexagonal hyper-space representations. During the course of osteogenic differentiation, the largest variations took place in 3 out of the 6 parameters, namely cell spreading area, magnitude of complex shear modulus and Col1A1 gene expression; specifically, these parameters increased by a factor of 9.8, 5, and 18, respectively. In contrast, for the case of adipogenic differentiation, the largest alterations were recorded in C/EBP, vinculin, and cell area, which changed by a factor of 1106, 0.1, and 8.4, respectively.

To focus on the differences between osteogenesis and adipogenesis, we further reduced the 6-D plot to a 2-D plot, with the relative magnitude of complex shear modulus (|G*| = [(G′)^2^ + (G″)^2^]^1/2^) along the horizontal axis, and the relative differentiation gene expression along the vertical axis (Fig. 6c). This plot reveals that the relative change in the magnitude of the complex shear modulus (|G*|), in conjunction with that in Col1A1 gene expression parameter (represented by the “vector” from Day 0 to Day 7 for osteogenic differentiation in Fig. 6c) may serve as a metric to track the osteogenic differentiation, to potentially forecast the trend (of osteogenic differentiation) within a few days after the induction. Likewise, the relative change in C/EBP gene expression, in conjunction with that of complex shear modulus (|G*|), (represented by the “vector” from Day 0 to Day 7 for adipogenic differentiation in Fig. 6c), can be monitored to forecast the adipogenic differentiation. The 2-D mapping, as depicted in Fig. 6c, helps to visualize and to compare quantitatively the progressing rate of the two trends of differentiation. A calculation of the Pearson correlation coefficients between (|G*|) and the gene expressions of Col1A1 (for the case of osteogenic differentiation) and of C/EBP (for the case of adipogenic differentiation) shows a strong positive correlation (with Pearson Correlation Coefficient PCC = 0.86) between (|G*|) and the gene expression of COL1A1 in osteogenic differentiation. In contrast, a strong negative correlation (PCC = −0.94) exists between |G*| and the gene expression of C/EBP during adipogenic differentiation.

This approach, i.e., mapping of critical biophysical and biochemical markers in a parametric space as is illustrated in this work may also serve to assess the efficiency of MSCs differentiation into other cell types.

## Discussion

In the present study, we show that the alterations of viscoelastic properties of hMSCs are drastically different in osteogenic and adipogenic differentiation. The recorded evolution of the intracellular shear moduli of MSCs during osteogenesis revealed that the cells became stiffer and more viscous (both G′ and G″ increased) and converted from viscous-like (G′ < G″) to elastic-like (G′ > G″) response. In contrast, the intracellular mechanical behavior remained viscous-like during adipogenic differentiation, while the cells became softer and less viscous (both G′ and G″ decreased) as they matured during differentiation. These changes were associated with stress fiber arrangement and formation. Prior to osteogenic differentiation, hMSCs possessed long and thin actin filaments which were fairly well-aligned along the cells major axis; in the course of osteogenic differentiation, the actin filaments became thicker and were distributed rather uniformly along different directions (Fig. 4). In contrast, the adipogenic differentiation process was concomitant with a loss of stress fibers and less actin alignment^18^. Similar trends of F-actin organization were also observed when stem cell lineage specification was directed by extracellular matrix elasticity^32^.

Contradictory to our results, McAndrews *et al*.^18^ reported that elastic modulus G′ of MSCs in adipogenic induction medium for 7 days was higher than that of MSCs in maintenance medium when cells were cultured on soft substrates (2 kPa and 8 kPa). We speculate that the differences between our results and those of McAndrews *et al*. can be attributed to one or more of the following factors: (1) Our VPTM measurements of differentiating MSCs were on rigid glass-bottom culture dishes (rather than on soft substrates); the effect of substrate stiffness on viscoelastic properties of differentiating cells has not been reported and warrants further investigations. (2) McAndrews *et al*. injected fluorescent particles before the chemical induction and the particles were retained inside MSCs during adipogenic differentiation for 7 days, whereas we injected fluorescent particles one day before each VPTM measurement. We speculate that the differentiation could be perturbed by the presence of the injected fluorescent particles. (3) We measured elastic modulus (G′) of undifferentiated MSCs (day 0) as well as differentiating MSCs on day 7, 14, 21 and 28 after the induction; hence, our results allow us to delineate the alteration of elastic modulus along the course of adipogenic differentiation at the time points far beyond the initial period of the differentiation. (4) McAndrews *et al*. report G′ and G″ measurements based on particle tracking at relatively low frequencies (0.1 to 10 Hz). VPTM measurements in the low frequency regime (~0.1 Hz to 1 Hz) of that frequency range are known to be contaminated by active processes that break down the assumption that the particles undergo equilibrium Brownian motion^33^.

The viscoelastic properties of the cell influence the transmission of mechanical forces throughout cellular domains, and modulate the amount and rate of cellular deformation in response to these forces. Our results imply that the changes in viscoelastic properties during differentiation are related to the dynamics of the cytoskeleton and focal adhesions. Cadherins are adhesion molecules involved in mechanotransduction and the formation of cadherin adhesions is correlated with the transmission of forces across neighboring cells^34^,^35^,^36^. Vinculin, a cytoskeletal and focal adhesion protein, has been reported to regulate stem cell fate in a force-dependent manner^37^, its phosphorylation has been related to force transmission^38^ and anchorage of vinculin to cell membranes to regulate the cell stiffness has been reported^39^. Tropomyosin is another actin-binding protein and functions as a regulator of calcium-dependent interaction of actin and myosin. A recent study indicated that tropomyosin, rather than actin itself, has direct impacts on cell stiffness in a tropomyosin isoform-specific manner^40^. In addition, vimentin, a widely distributed intermediate filament protein which exhibits viscoelastic properties that are distinct from those of actin, is suggested to play an important role in maintaining mechanical integrity of cells^41^,^42^,^43^. Consistently, our results show that expressions of these four genes increase during osteogenesis. However, during adipogenesis cadherin and vimentin were stably expressed while the expressions of vinculin and Tropomyosin alpha-1 (TPM1) were downregulated (Fig. 1). Vimentin plays important roles in various cellular functions including wound healing, migration, mechanotransduction, as well as acting as signaling organizer^44^. It has been shown that the expression and organization of vimentin affects phosphorylation of Rho or ERK1/2 mediated signaling^45^,^46^, which is related to osteogenesis^47^,^48^. Therefore, the tendency of increasing vimentin expression during osteogenic differentiation may contribute to both osteogenic related phosphorylation signaling and elastic-like behavior. However, since mechanosensitive vinculin does not play structural roles in the formation of focal adhesion complex^37^, we hypothesize that tropomyosin upregulation stiffens the cytoskeleton and thereby increasing forces at vinculin, which was also upregulated during osteogenesis, for mechanotransduction; this positive feedback loop could further contribute to the increase in intracellular viscoelasticity.

The optimized seeding densities we used for osteogenic and adipogenic differentiations were 40% and 100%, respectively. Since cell-cell adhesion mediator N-cadherin is suggested to be associated with the alteration of viscoelasticity (Fig. 1c), we therefore further investigated whether cell-cell contact could affect the alteration of viscoelasticity. Our results show that gene expression patterns of Runx2 and Vimentin were unaffected when seeding density was increased from 40% to 100% in osteogenic differentiation; even though cell-cell contacts suppressed the expressions of ColIA1, TPM1, CDH2, and vinculin in early stages, the gene expressions resumed to the same level as the control 21 days after the induction. Consistent with those of differentiating MSCs induced at 40% confluency, both G′ and G″ gradually increased after the osteogenic induction with the ratio of G′/G″ reaching approximately the same level as the control 14 days after the induction (Supplementary Fig. S4). In the case of high seeding density, we observed early maturation in mineralization on day 21; the mineralized matrix deposition hinders carboxylated polystyrene particles from entering (via injection) into the cells; hence, the viscoelastic parameters could not be measured accurately after day 21. For the terminal differentiated cells, both extracellular calcium deposition and lipid droplets could hinder fluorescent particles injection into the cells; hence, we may overlook terminally differentiated cells while performing VPTM measurement. Differentiating MSCs give rise to a slightly heterogeneous population; in the case of osteogenic differentiation, the terminal differentiated cells contain intracellular calcium deposition which may also affect viscoelastic properties. The magnitudes of G′, G″, as well as G′/G″ tend to increase during the course of osteogenic differentiations; therefore possible under-sampling of those terminal differentiated cells at the late stage of differentiation may lead to an underestimation of the mean value of viscoelasticity. Nevertheless, the results with different seeding densities demonstrate that the alternation in viscoelastic properties can distinguish the differentiation direction regardless of the confluence.

By means of video particle tracking technique (VPTM), we have shown that in the course of osteogenic differentiation of MSCs, cells increased their intracellular stiffness and viscosity, and converted from viscous-like to elastic-like behavior, in concert with the ongoing biochemical changes and gene expressions. The changes in viscoelastic properties were accompanied by significant rearrangement of the actin cytoskeleton. In contrast, both the intracellular stiffness and viscosity of hMSCs decreased during adipogenic differentiation, and there were no significant changes in their viscous-like behavior or the arrangement of the actin cytoskeleton. The distinct alteration patterns of intracellular viscoelasticity between osteogenesis and adipogenesis, in conjunction with biochemical parameters based on genes expression and biophysical parameters, such as cell morphology, cell area and cytoskeleton arrangement, provide a unique approach to characterize the differentiation directions. In addition, this technique is well suited for the study of the mechanobiology of differentiating stem cells, especially in three dimensional biomimetic environments with different stiffness^49^ or with different chemical constituents such as blockers of actin polymerization^50^. A possible extension/modification of the analysis described in this paper is to use regression model to fit the experimental data obtained from hMSCs differentiation under one specific physical and chemical environment to predict and to validate experimentally the sensitivity to each of the parametric changes.

## Materials and Methods

### Culture and directed differentiation of human bone marrow–derived MSCs

Human mesenchymal stromal cells (hMSCs) were purchased from Steminent Biotherapeutics (Taipei, Taiwan) and maintained in Iscove’s Modified Dulbecco’s Medium (IMDM; Sigma−Aldrich) containing 10% fetal bovine serum (FBS; ES-qualified, Life Technologies), 10 ng/ml basic fibroblast growth factor (FGF2; Sigma-Aldrich), and 1% PSG (Sigma−Aldrich). Cells were seeded at the confluency of 30% to 40% and replated under the same culture conditions once reaching the confluency around 70%. hMSCs of 15^th^ passage were used for the experiments and cultured on Borosilicate glass-bottom dishes (Alpha Plus, Taiwan) coated with 100 μg/ml bovine type I collagen (Purecol, Advanced Biomatrix). For directed osteogenic differentiation, cells of 40% confluency were treated with the induction medium of IMDM supplemented with 100 nM dexamethasone, 10 mM β-glycerol phosphate, 0.2 mM ascorbic acid, and 1% PSG. The osteogenic induction medium was changed every 7 days. For directed adipogenic differentiation, confluent cells were treated with the induction medium of IMDM supplemented with 0.5 mM Isobutylmethylxanthine (IBMX), 1 uM dexamethasone, 100 nM Indomethacin, 10% FBS, and 1% PSG. The adipogenic induction medium was changed twice per week. The efficiency of directed differentiations was confirmed by Alizarin Red S staining for osteogenesis and Nile Red staining for adipogenesis (Supplementary Fig. S8).

### Quantitative real-time polymerase chain reaction

Total RNA was extracted by RNeasy Mini Kit (QIAGEN). Up to 1 μg of total RNA was reverse transcribed to complementary DNA by MMLV High Performance Reverse Transcriptase according to manufacturer’s instructions (EPICENTRE). Quantitative real-time PCR (qPCR) was performed with TaqMan^®^ Fast Universal PCR Master Mix (2X) by Step One plus real-time PCR system (Applied Biosystems) to determine the relative gene expression profiles. The primers used for qPCR are listed in Supplementary Table S1.

### Immunofluorescent staining

Primary antibody against vinculin (Abcam, UK) was used at 1:100 dilution. Nuclei and F-actin were stained with DAPI and phalloidin respectively. Immunofluorescent staining was performed according to the manufacturer’s instructions and the images were taken by Olympus FluoView™ FV1000 confocal microscope using the same settings of laser power and pinhole size.

### Video particle tracking microrheology

Video particle tracking microrheology (VPTM) was used to determine the intracellular viscoelasticity^51^,^52^,^53^. Human mesenchymal stem cells, at different differentiation stages in the course of differentiation from day 0 to day 28, were cultured on 35 mm collagen coated glass bottom culture dishes (α-PLUS), and were injected with 20 μL carboxylated polystyrene particles (Invitrogen, fluorescence excitation/admission peaks: 580 nm/605 nm, diameter = 200 nm, concentration: 3.9 × 10^8^ particles/μL) via a biolistic particle delivery system (PDS-100, Bio-Rad; pressure 450 psi). After particle injection, cells were washed with PBS twice and incubated in culture media. After overnight of incubation, cells were placed in a living-cell incubator chamber (Tokai Hit, Shizuoka-ken, Japan) of an inverted fluorescence microscope (Nikon Eclipse Ti), equipped with an oil immersion objective (Nikon, 100X/N.A = 1.45) and a CMOS camera (Hamamatsu, ORCA-Flash 4.0) to record the motion of intracellular particles for 5 sec. with a frame rate of 100 frames per sec. and an image resolution of 0.13 μm/pixel (with binning 2 setting). The two-dimensional (transverse) projection of the Brownian motion of intracellular particles were tracked and analyzed via customized Matlab programs. The ensemble–averaged mean squared displacement (MSD), <r^2^(τ)> = <[x(t + τ) − x(t)]^2^ + [y(t + τ) − y(t)]^2^>, where τ is the time lag and t is elapsed time, was calculated from two-dimensional trajectory, x(t) and y(t), of each particle. The intracellular viscoelasticity in term of elastic modulus (G′) and viscous modulus (G″) were deduced from MSD based on a pseudo-Stokes Einstein equation^22^,^54^. In our VPTM system, the Brownian motion of ~10 to 30 particles per cells (Supplementary Video and Supplementary Fig. S9) was recorded in the field of view of a 100× (NA = 1.45) objective lens, in conjunction with a high-speed (100 frames per sec.) and high space-bandwidth product (1024 × 1024 pixels) CMOS camera with sub-micron spatial resolution. In each setting, a sequence of time-lapse images was recorded for 5 sec at a frame rate of 100 frames per sec, which enabled us to deduce the intracellular elastic and viscous moduli as a function of frequency “*f *” (in the range of ~0.2 to 100 Hz.). We restrict our analysis to the frequency range of ~1 to 100 Hz (see Supplementary Fig. S3) to avoid the low-frequency artifacts introduced by potential cell motions and insufficient statistical convergence.

### Image analysis

For the analysis of cell stress fibers orientation and FAs, hMSCs, at different stages of differentiation, were stained with FITC labeled-phalloidin and RFP labeled vinculin. The images were captured via a Zeiss LSM 700 confocal microscope with a 40× oil-immersion objective (NA = 1.4). The orientation of stress fibers were analyzed by customized Matlab programs^26^. We used previously described image processing methods to segment F-actin fibers and quantify their orientation angles^53^,^55^,^56^,^57^ (Supplementary Fig. S10). The algorithm thresholds fluorescence images of F-actin to create binary images containing segmented fibers. These binary images were convolved with linearly-varying kernels in the x and y directions to obtain smooth maps of the two components of the image intensity gradient, G_x_ and G_y_. Then, the local orientation of the fibers was obtained as 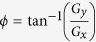 The segmented fibers were color-coded to represent the orientations of the fibers, and their angular distribution is presented as shown in Supplementary Fig. S10. The individual FAs area and number of FAs per cells were determined and analyzed by MetaMorph software. The cellular morphology was characterized in terms of cell area and cell aspect ratio (the ratio of the length of major to minor axes) via customized Matlab programs.

### Statistical analysis

Statistical significance of the experimental results was evaluated by a two-tailed Student’s t-test. Significant differences are designated by * for *P* < 0.05 and ** for *P* < 0.01. Statistically significant differences of angular distributions of stress fibers at different stages of differentiation were obtained by Watson’s U^2^ test.

## Additional Information

**How to cite this article**: Chen, Y.-Q. *et al*. Bio- chemical and physical characterizations of mesenchymal stromal cells along the time course of directed differentiation. *Sci. Rep.* **6**, 31547; doi: 10.1038/srep31547 (2016).

## Supplementary Material

Supplementary Information

Supplementary Video
