# Supplementary material for: Bio- chemical and physical characterizations of mesenchymal stromal cells along the time course of directed differentiation
Source: Sci Rep. 2016 Aug 16;6:31547. doi: 10.1038/srep31547 (PMC4985743; doi:10.1038/srep31547)
Supplement: Supplementary Information [file srep31547-s1.doc]

**Supplementary Materials for**

**Bio- chemical and physical characterizations of mesenchymal stromal cells along the time course of directed differentiation**

Yin-Quan Chen, Yi-Shiuan Liu, Yu-An Liu, Yi-Chang Wu, Juan C. del Álamo, Arthur Chiou, Oscar K. Lee


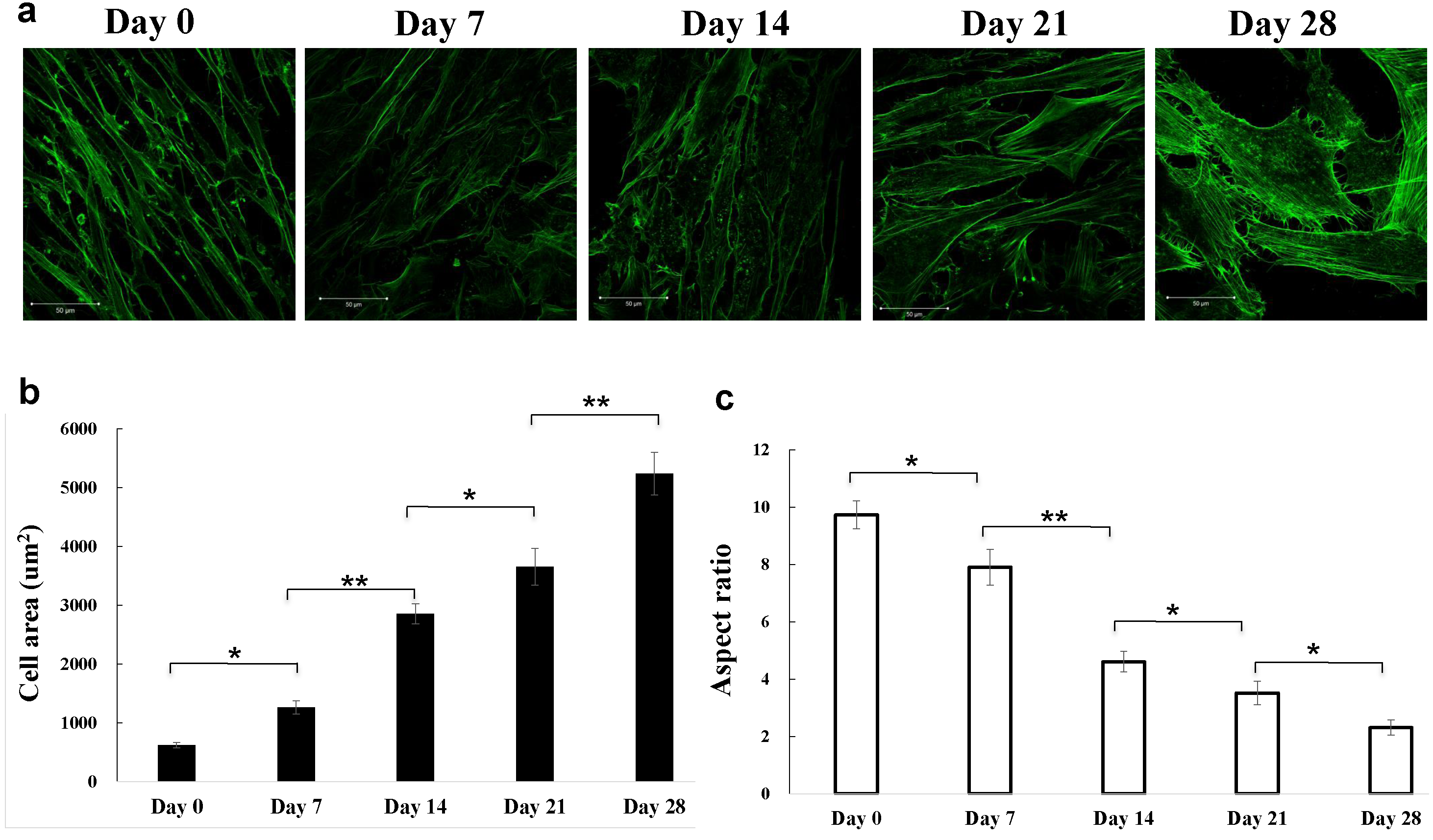


**Fig. S1. Cellular morphological changes during the course of adipogenic differentiation. (a)** Immunofluorescence micrographs of actin filaments of hMSCs during adipogenic differentiation. Quantification of **(b)** cell spreading area, and **(c)** aspect ratio (defined as the ratio of the length of major and minor axes) at different stages of adipogenic differentiation of hMSCs. The mean values and standard error of the mean (SEM) are indicated by the height of the thick bars and the thin lines. *: *p* < 0.05; **: *p* < 0.01 (by Student’s t-test). Data obtained from N = 15 cells.

**
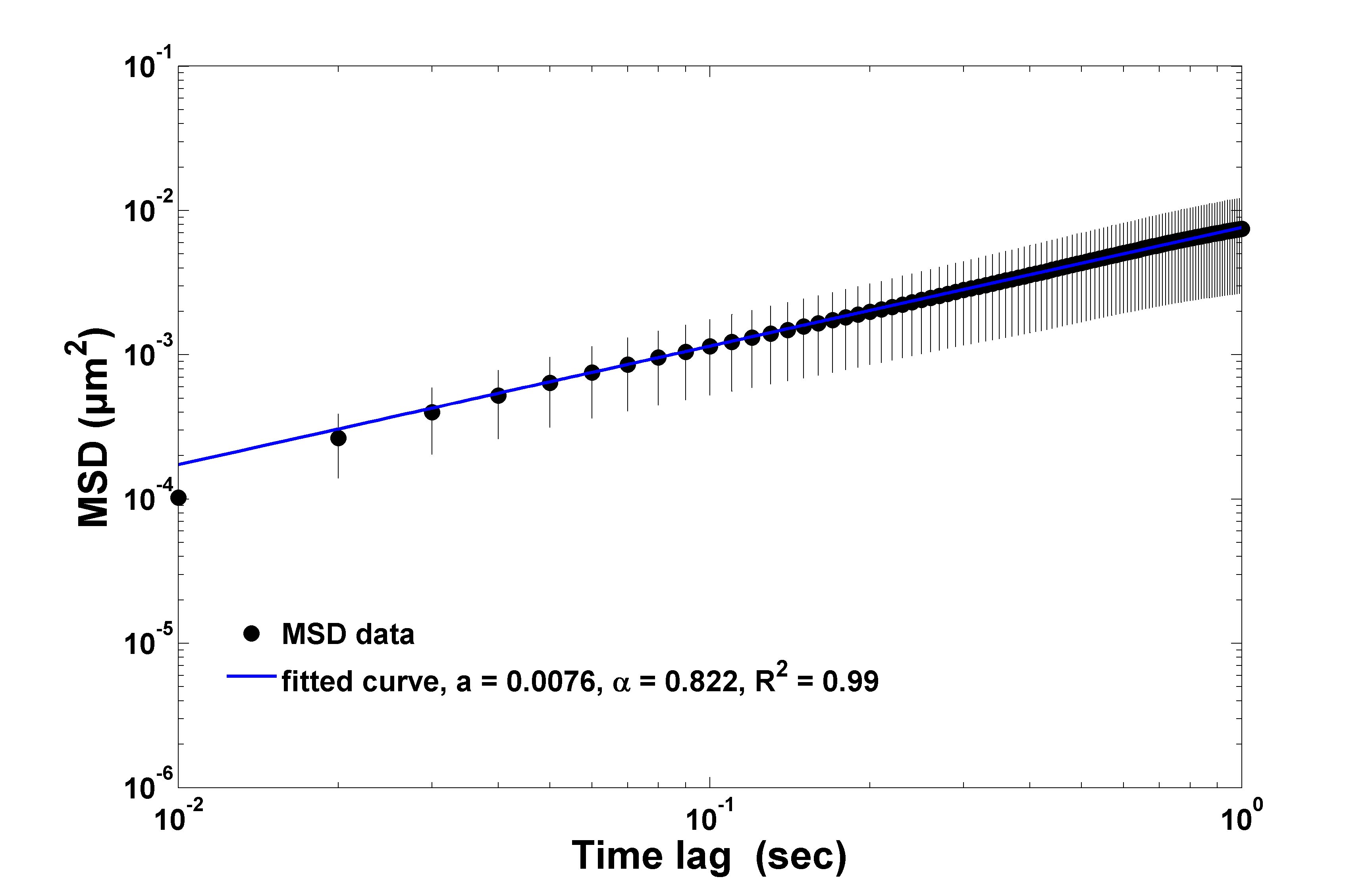
**

**Fig. S2. The mean squared displacement (MSD) as a function of the time lag (τ) of hMSCs at day 0.** Data (obtained from 404 beads in 30 cells) are expressed as mean ± SD. The straight line represents the power law dependence [**MSD = a**τ**^α^**] of the best fit with two fitting parameters a = 0.0076 and *α* = 0.822.


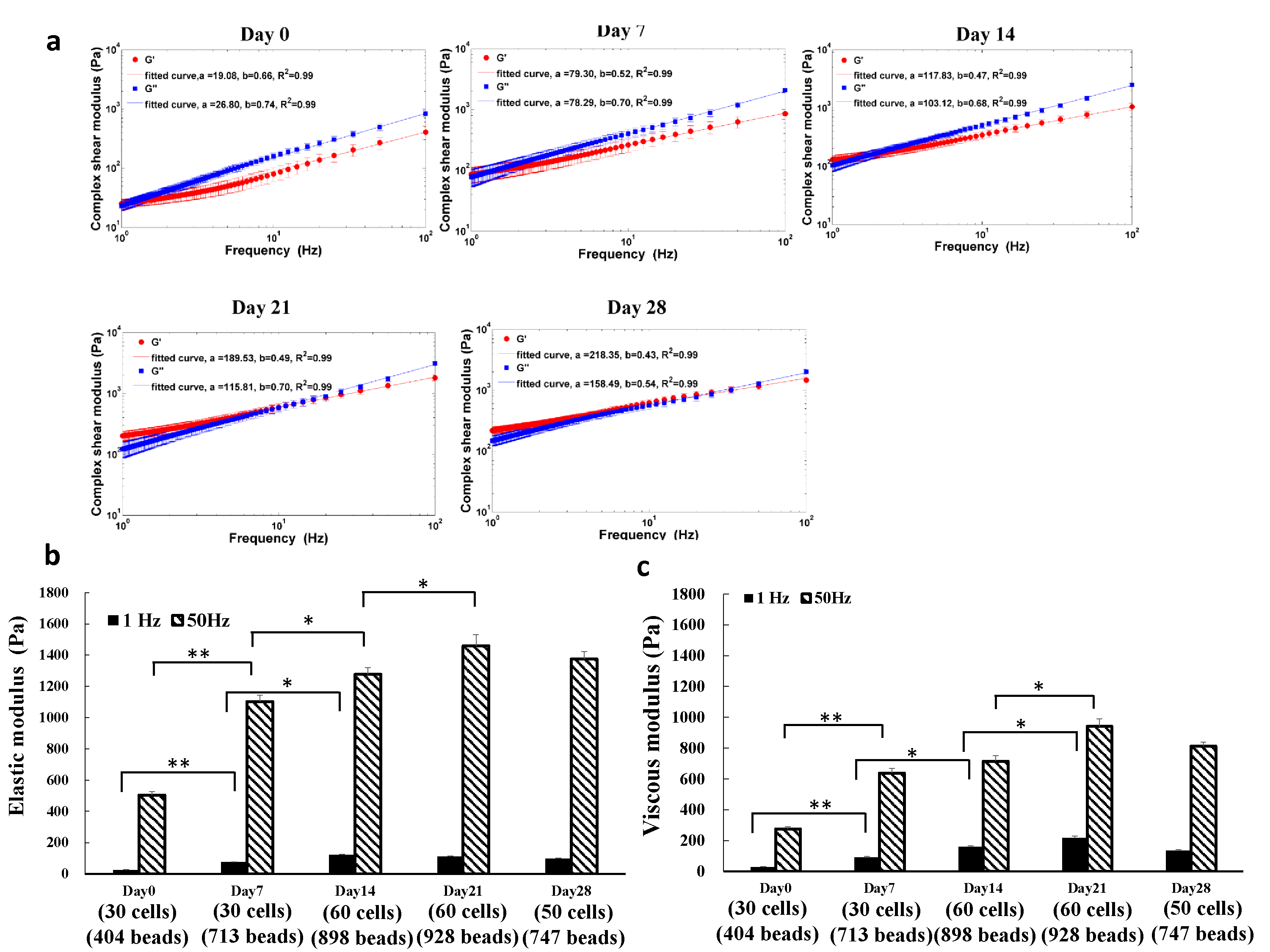


**Fig. S3. Intracellular viscoelasticity of hMSCs during osteogenic differentiation from Day 0 to Day 28 at 7-day interval. (a**) The intracellular elastic modulus (G') and viscous modulus (G") of hMSC as function of frequency. The straight lines represent the power law dependence; the symbols represent the mean value of the experimental results. The vertical bars around each point represent the corresponding standard error of the mean. **(b)** The intracellular elastic modulus (G′) and **(c)** viscous modulus (G″), at 1Hz and 50Hz, of hMSCs during osteogenic differentiation. *: *p* < 0.05; **: *p* < 0.01 (by Student’s t-test).


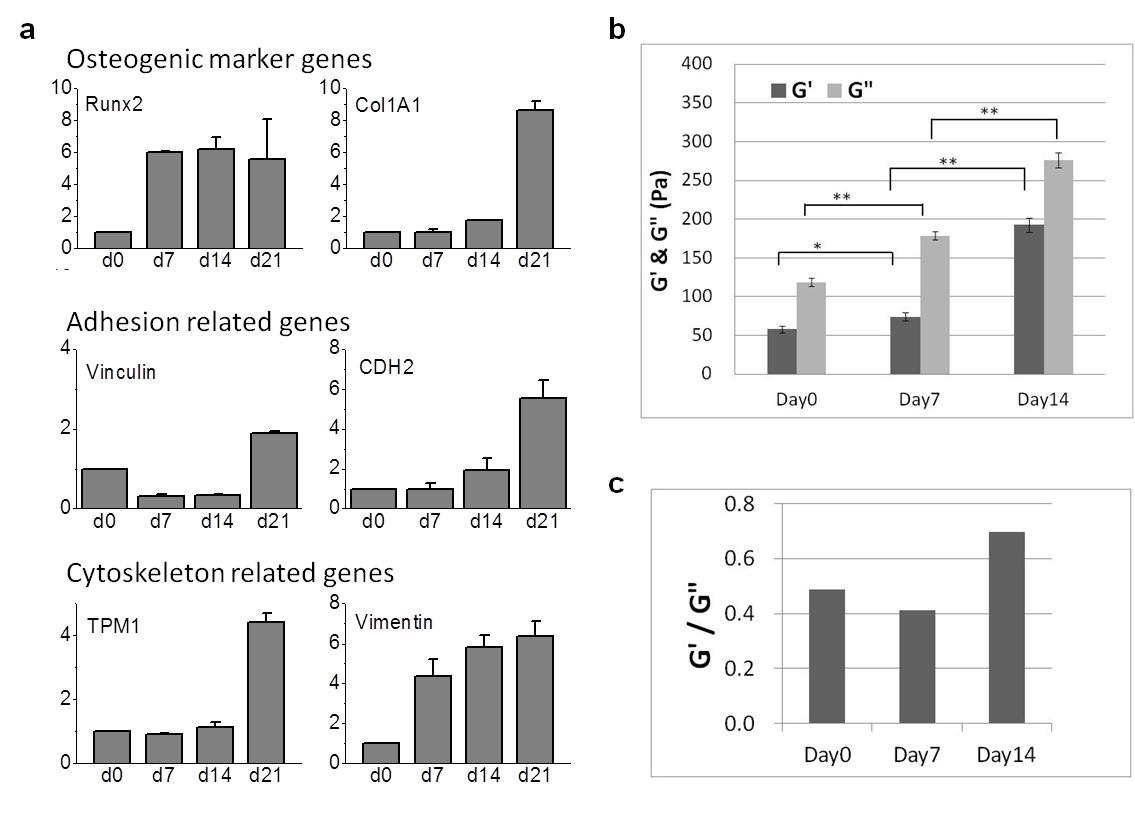
**Fig. S4. Gene expression profile and the change in viscoelasticity of confluent hMSCs during the course of osteogenic differentiation. (a)** Relative gene expressions of osteogenic marker genes Runx2 and collagen type 1 alpha 1, adhesion related genes Vinculin and CDH2, as well as cytoskeleton related genes TPM1 and Vimentin in osteogenic differentiation when hMSCs were induced upon confluence for osteogenesis. Gene expressions were analyzed by RT-PCR. Data were normalized by the respective gene expressions of undifferentiated MSCs (day 0) and presented as mean ± SD. **(b)** The intracellular elastic modulus (G′) and viscous modulus (G″) (at 10Hz) of hMSCs during osteogenic differentiation. Error bars represent SEM. *: *p* < 0.05; **: *p* < 0.01. **(c)** Ratio of G′ to G″ from day 0 to day 14 after the induction.


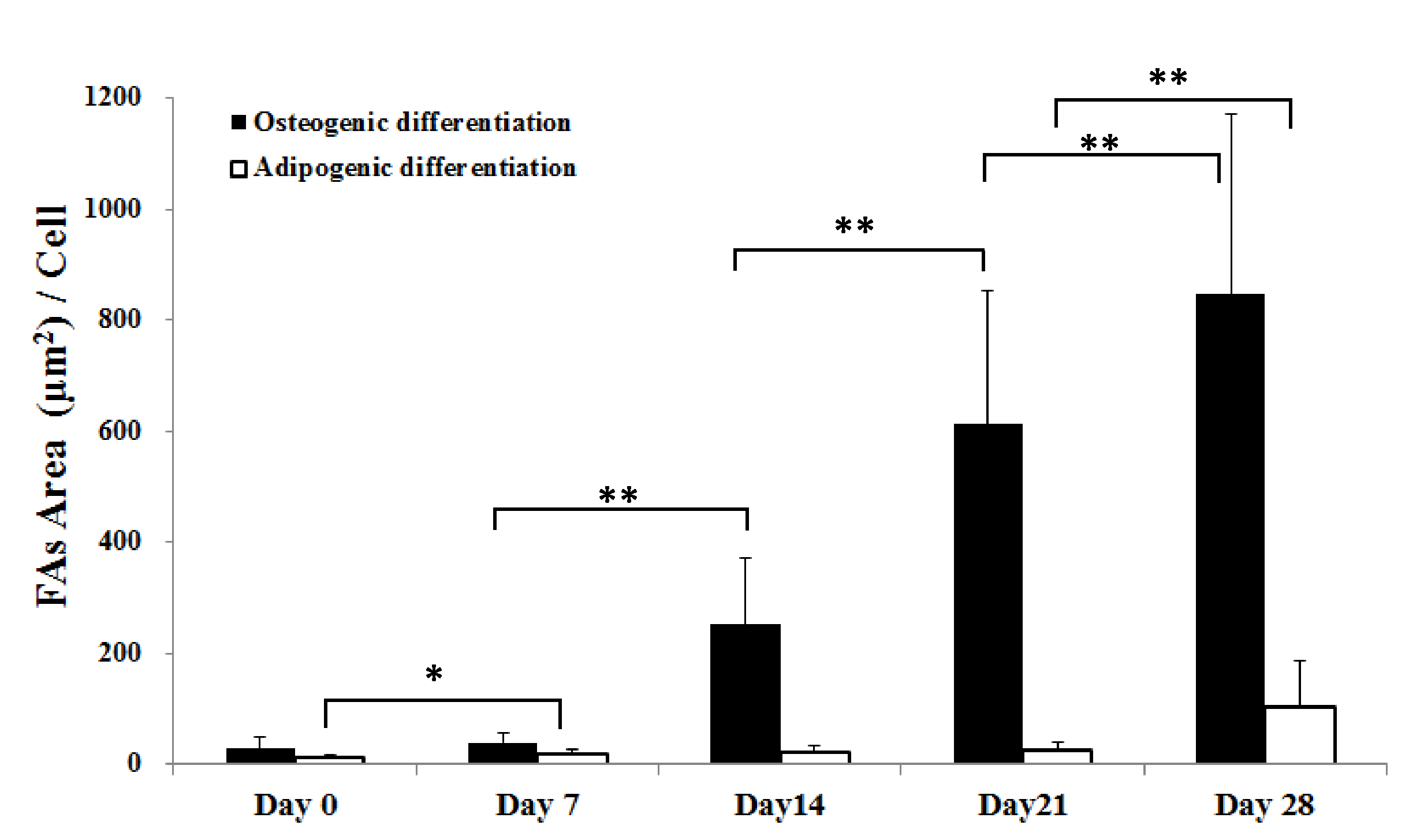


**Fig. S5. Change in total focal adhesion area during the time course of osteogenic differentiation and adipogenic differentiation.** All data (obtained from 15 cells) are expressed as mean ± SD. *: *p* < 0.05; **: *p* < 0.01 (by Student’s t-test).


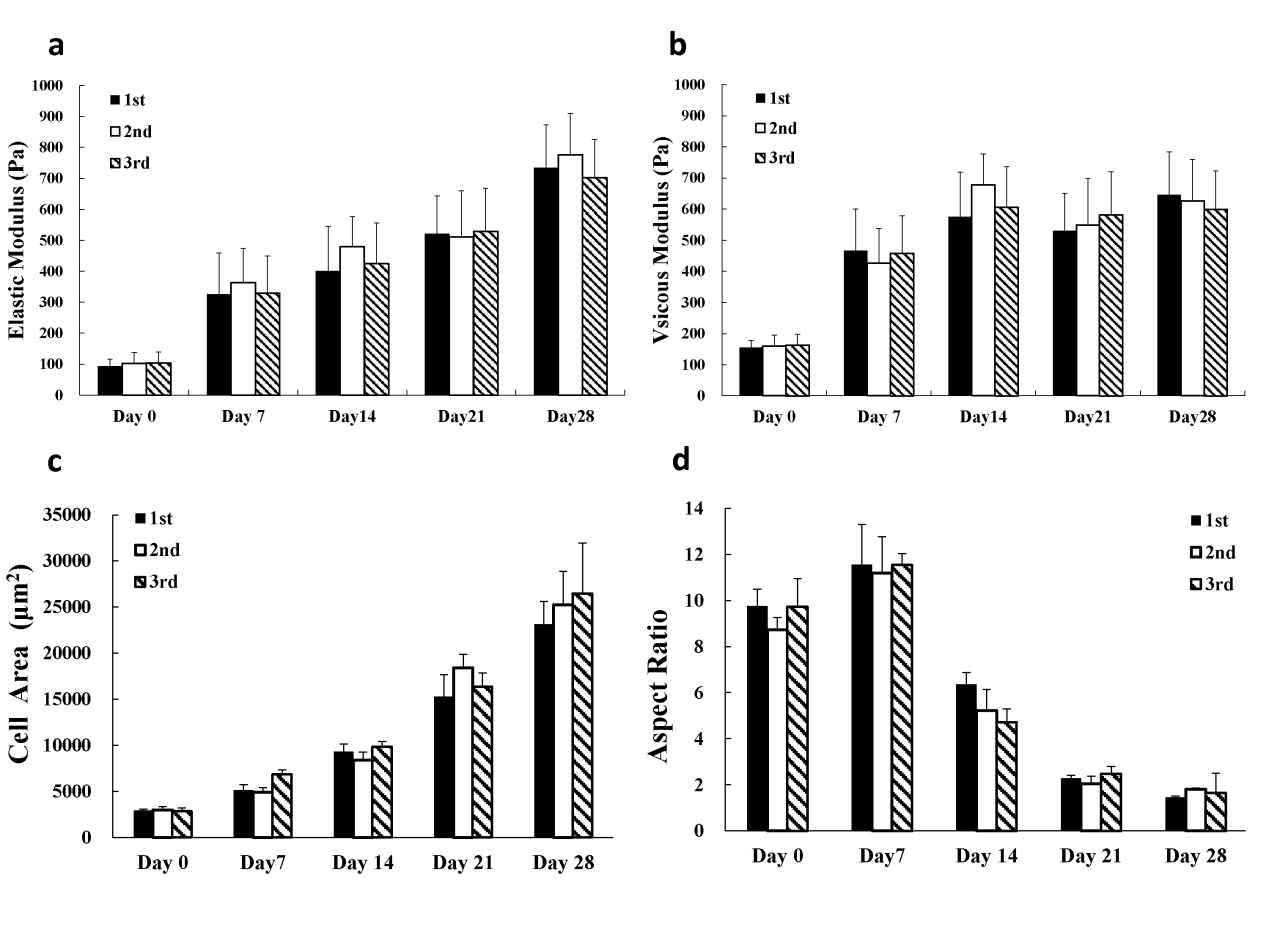


**Figure S6**. **Intracellular viscoelasticity and cellular morphological changes of hMSCs during osteogenic differentiation. (a)** Intracellular elastic modulus (G′), **(b)** viscous modulus (G″), both at 10Hz, **(c)** cell spreading area and **(d)** aspect ratio (defined as the ratio of the length of major and minor axes) of hMSCs during osteogenic differentiation for three replicate experiments with 30 cells per replicate. The bar charts represent mean ± S.E.M (standard error of the mean).


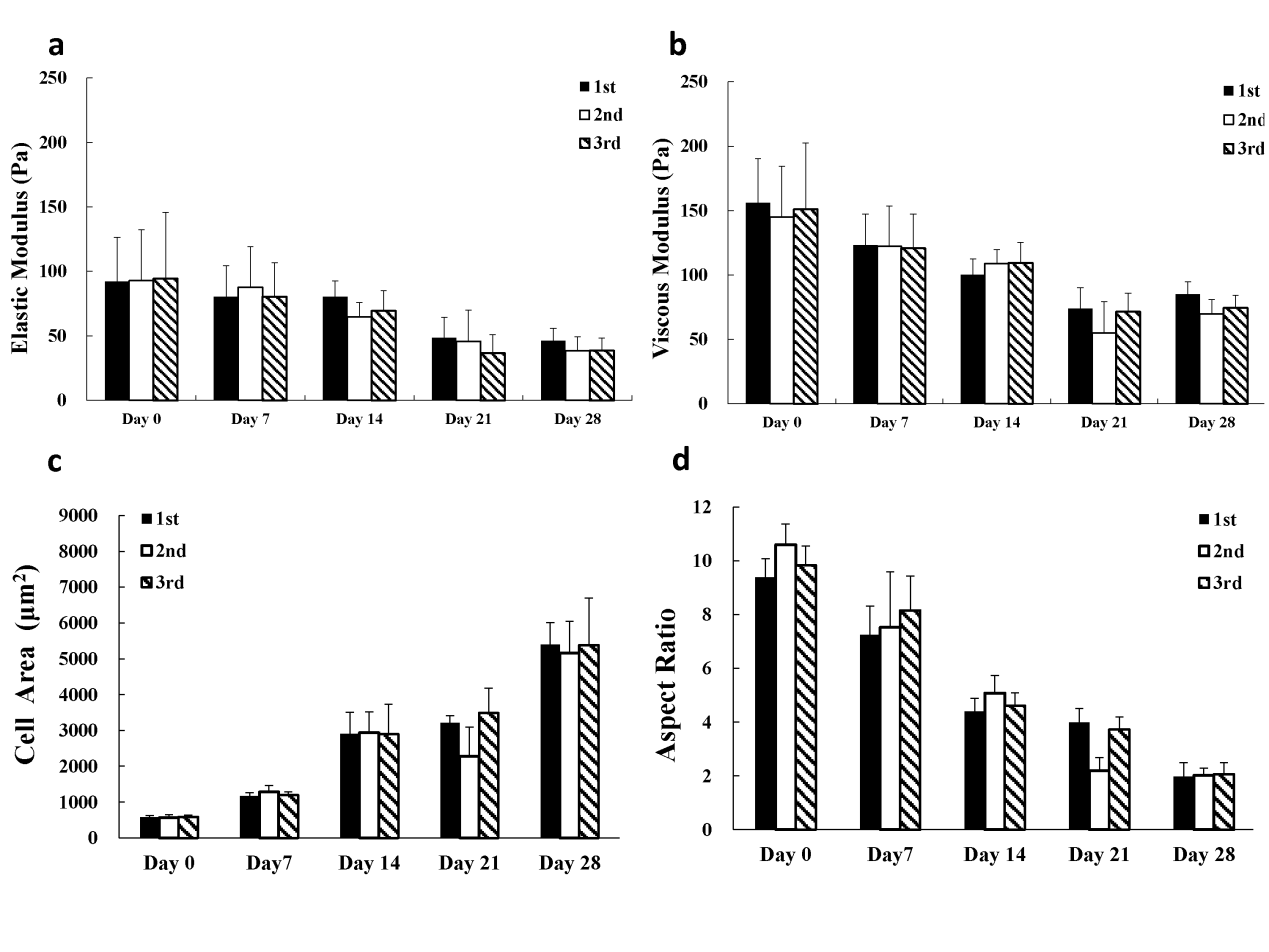


**Figure S7**. **Intracellular viscoelasticity and cellular morphological changes of hMSCs during adipogenic differentiation. (a)** Intracellular elastic modulus (G′), **(b)** viscous modulus (G″), both at 10Hz, **(c)** cell spreading area and **(d)** aspect ratio (defined as the ratio of the length of major and minor axes) of hMSCs during adipogenic differentiation for three replicate experiments with 30 cells per replicate*.* The bar charts represent mean ± S.E.M (standard error of the mean).

**a**


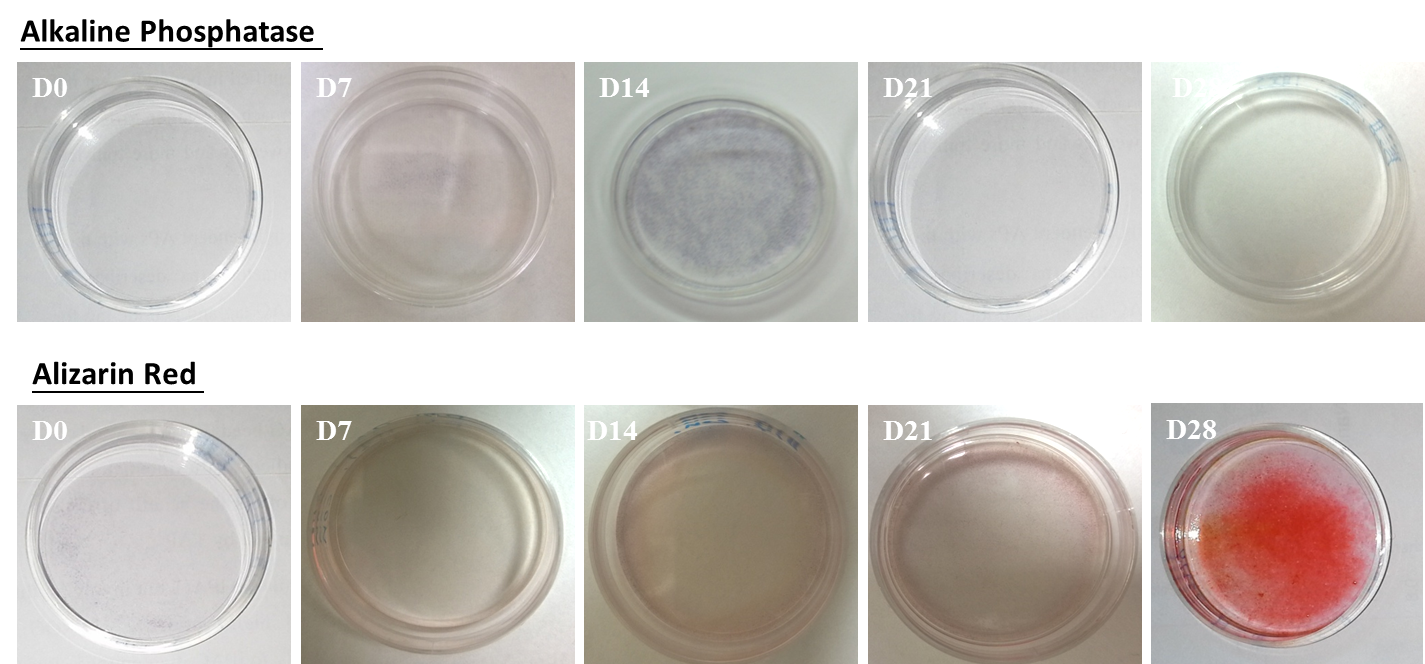


**b**


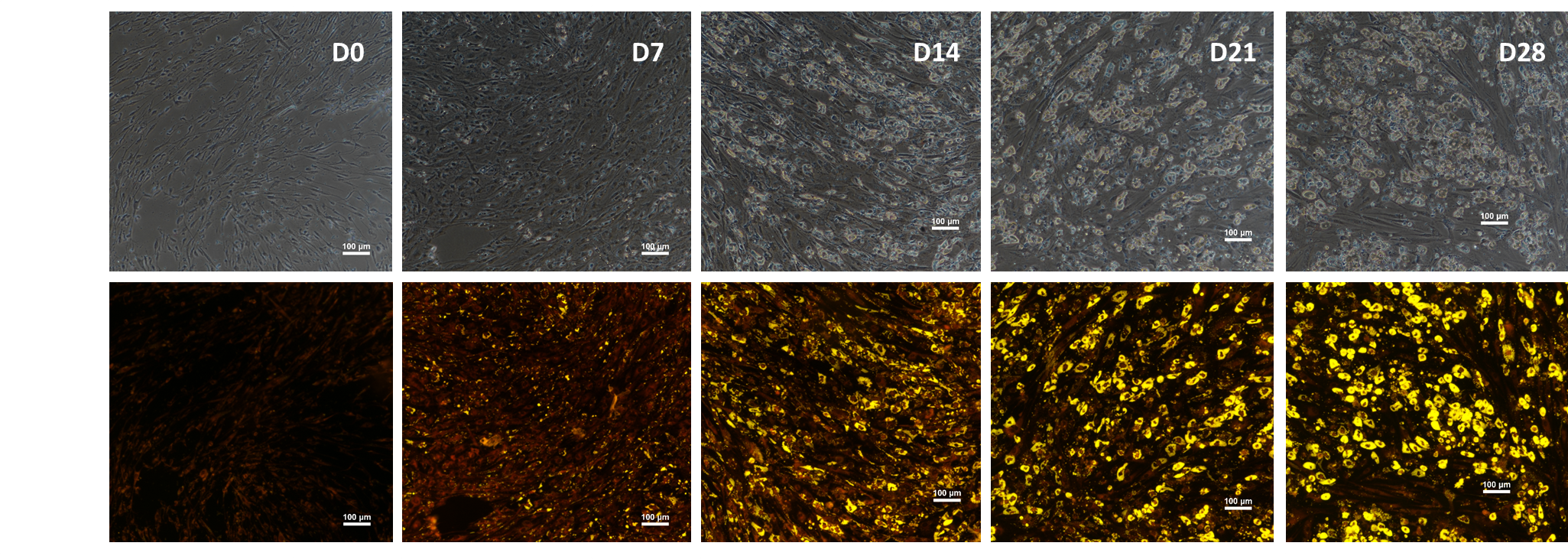


**Fig. S8.** **The histological images of osteogenic and adipogenic differentiations over differentiation time course.** **(a)** Alkaline Phosphatase and Alizarin Red S staining for osteogenic differentiation. **(b)** Bright field images and Nile Red staining (50 msec exposure time for all fluorescent images) during adipogenic differentiation. MSCs were first fixed with 3.7% formaldehyde (Sigma−Aldrich) and washed with phosphate-buffered saline (PBS). Fixed cells were then incubated with 5-bromo-4-chloro-3-indolyl phosphate/nitro blue tetrazolium (BCIP/NBT, Sigma−Aldrich) in dark for 1 hour to examine Alkaline phosphatase (ALP) activity, or with Nile Red 4 μg/ml (Sigma−Aldrich) in dark for 20 minutes to localize lipid droplets within cells. Samples were imaged by inverted microscope immediately after rinsed in PBS. For Alizarin Red S staining, fixed cells were washed by distilled water and stained with Alizarin Red solution (2% w/v, pH 4.1, Sigma−Aldrich) for 5 minutes. Samples were then rinsed in distilled water again and imaged by inverted microscope.


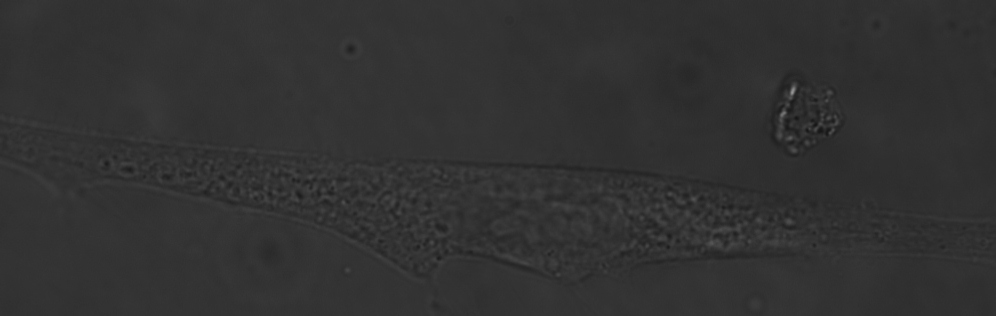


**Fig. S9.** The phase contrast image of single hMSCs corresponding to the fluorescence movie of a hMSC cell with injected fluorescent beads (fluorescence excitation/admission peaks: 580 nm/605 nm, diameter = 200 nm, Supplementary Video).


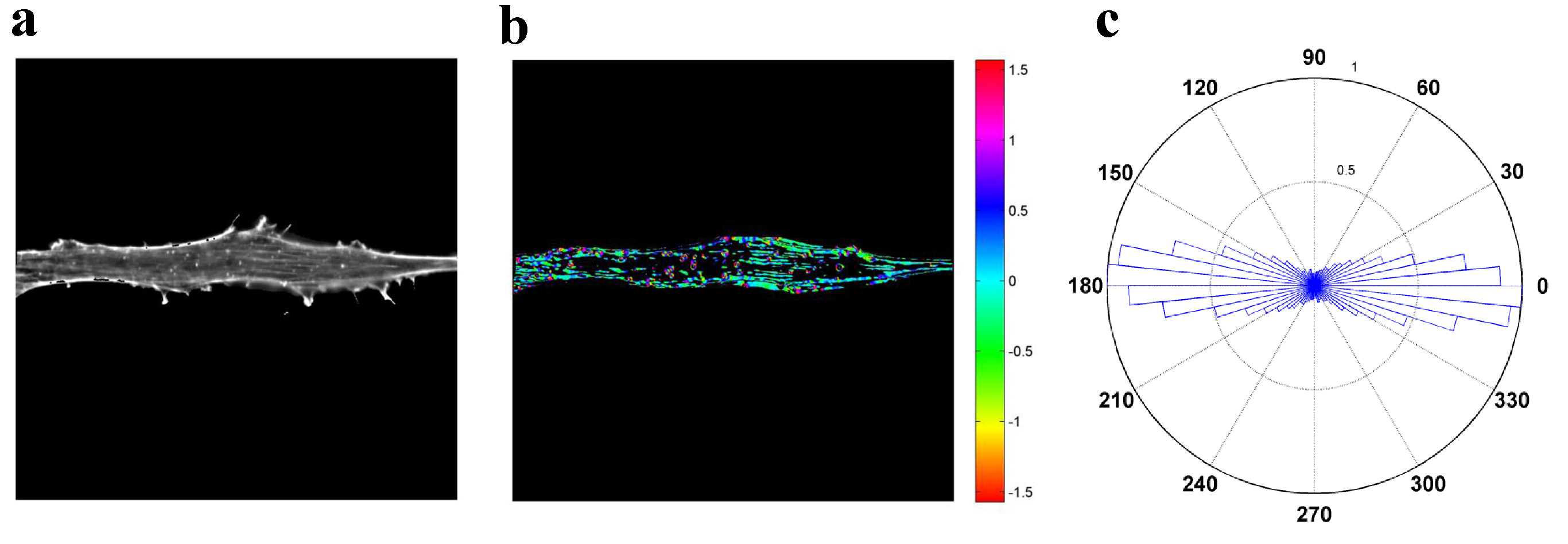


**Fig. S10. The F-actin orientation in the basal region of hMSCs at day 0.** **(a)** A micrograph of a cell fixed and stained with FITC-phalloidin (green) for F-actin. **(b)** The image of actin fibers with fluorescence intensity above a selected threshold were marked and color-coded to represent their angular orientation in radians; the direction along the principal axis (i.e., the direction along which the maximum number of actin filaments were aligned) is denoted as 0. **(c)** The histogram of F-actin orientation corresponding to (b).

## Table 1. Sequences of RT-PCR primers for hMSCs

| **Gene** | **Sequences** | **Probe** |
| --- | --- | --- |
| **GAPDH** | agccacatcgctcagacac / gcccaatacgaccaaatcc | 60 |
| **Osterix** | gactgcagagcaggttcctc / taacctgatggggtcatggt | 43 |
| **Runx2** | gtgcctaggcgcatttca / gctcttcttactgagagtggaagg | 29 |
| **Osteonectin** | gtgcagaggaaaccgaagag / tgtttgcagtggtggttctg | 77 |
| **Collagen type I A1** | gggattccctggacctaaag / ggaacacctcgctctcca | 67 |
| **Adiponectin** | ggtgagaagggtgagaaagga / tttcaccgatgtctcccttag | 85 |
| **PPARG** | tccatgctgttatgggtgaa / tgtgtcaaccatggtcatttc | 14 |
| **GLUT4** | ctgtgccatcctgatgactg / cgtagctcatggctggaact | 67 |
| **C/EBPα** | GACATCAGCGCCTACATCG / GGCTGTGCTGGAACAGGT | 72 |
| **CDH2** | ctccatgtgccggatagc / tctacagacgcctgaagcag | 74 |
| **Vinculin** | gatgaagctcgcaaatggtc / tctgcctcagctacaacacct | 28 |
| **Vimentin** | ttcgccaactacatcgacaa / cggccagcaggatcttatt | 1 |
| **TPM1** | agcgtctggcaacagctt / gggctcgactctcaatgact | 65 |
